# Mandibular jaw movements as a non-invasive measure of respiratory effort during sleep: application in clinical practice

**DOI:** 10.3389/frsle.2023.1145620

**Published:** 2023-05-12

**Authors:** Jean-Benoit Martinot, Jean-Louis Pépin

**Affiliations:** ^1^Sleep Laboratory, Centre Hospitalier Universitaire (CHU) Université catholique de Louvain (UCL) Namur Site Sainte-Elisabeth, Namur, Belgium; ^2^Institute of Experimental and Clinical Research, Université catholique de Louvain (UCL) Bruxelles Woluwe, Brussels, Belgium; ^3^HP2 Laboratory, Inserm U1300, University Grenoble Alpes, Grenoble, France

**Keywords:** mandibular jaw movements, obstructive sleep apnea, respiratory effort, home sleep apnea test (HSAT), respiratory drive, automated analysis

## Abstract

Assessment of respiratory effort (RE) is key for characterization of respiratory events. The discrimination between central and obstructive events is important because these events are caused by different physio-pathological mechanisms and require different treatment approaches. Many of the currently available options for home sleep apnea testing either do not measure RE, or RE signal recording is not always reliable. This is due to a variety of factors, including for instance wrong placement of the respiratory inductance plethysmography (RIP) sensors leading to artifacts or signal loss. Monitoring of mandibular jaw movements (MJM) provides the ability to accurately measure RE through a single point of contact sensor placed on the patient's chin. The inertial unit included in the capturing technology and overnight positional stability of the sensor provide a robust MJM bio-signal to detect sleep-disordered breathing (SDB). Many of the pharyngeal muscles are attached to the mandible directly, or indirectly via the hyoid bone. The motor trigeminal nerve impulses to contract or relax these muscles generate discrete MJM that reflect changes in RE during sleep. Indeed, the central drive utilizes the lower jaw as a fine-tuning lever to stiffen the upper airway musculature and safeguard the patency of the pharynx. Associations between the MJM bio-signal properties and both physiological and pathological breathing patterns during sleep have been extensively studied. These show a close relationship between changes in the MJM bio-signal as a function of RE that is similar to levels of RE measured simultaneously by the reference bio-signals such as esophageal pressure or crural diaphragmatic electromyography. Specific waveforms, frequencies, and amplitudes of these discrete MJM are seen across a variety of breathing disturbances that are recommended to be scored by the American Academy of Sleep Medicine. Moreover, MJM monitoring provides information about sleep/wake states and arousals, which enables total sleep time measurement for accurate calculation of conventional hourly indices. The MJM bio-signal can be interpreted and its automatic analysis using a dedicated machine learning algorithm delivers a comprehensive and clinically informative study report that provides physicians with the necessary information to aid in the diagnosis of SDB.

## Introduction

The American Academy of Sleep Medicine (AASM) scoring rules for sleep-related respiratory events are based on changes in airflow captured by dedicated sensors and associated with an arousal and/or oxygen desaturation. Another key physiologic parameter required to assess sleep-disordered breathing (SDB) is respiratory effort (RE) to allow differentiation between central and obstructive apneas; while increased RE reflects obstructive SDB events, which are the most frequent breathing disturbances, decreased RE is mandatory for a reliable scoring of central events including hypopneas (Berry et al., 2012, 2017).

The gold standard marker of RE during sleep is the amplitude of the esophageal pressure (PES) curve, a surrogate for diaphragmatic muscular contraction in the presence of increased flow resistance in the upper airway. However, esophageal manometry is an invasive method that is rarely used in clinical practice because of associated patient discomfort and related sleep alterations (Vandenbussche et al., 2015). Failure to correctly detect increased RE when present may result in incorrect classification of breathing disturbances and contribute to incorrect therapeutic decision making (Randerath et al., 2013; Martinot et al., 2019; Randerath, 2021). In addition, the high and rising prevalence of obstructive sleep apnea (OSA) highlights the need for reliable home sleep apnea testing (HSAT) options (Raphelson et al., 2022). However, measurement error compared with conventional polysomnography (PSG) can be problematic with HSAT. This is due to several factors, including inappropriate placement of sensors, use of recording time rather than sleep time as a denominator for calculation of SDB indices, technical issues leading to failed studies, and many others as previously summarized (Malhotra et al., 2015). Also, some specific clinical conditions, including associated cardiovascular comorbidities that favor central events, can preclude performance of HSAT.

This article details the use of a new-generation bio-signal, mandibular jaw movements (MJM), easily captured using a connected device set up on the chin by the patient at home, and recently validated against the gold standard measurement of RE, i.e., PES monitoring (Pépin et al., 2022).

## PSG evaluation of normal respiratory activity of the mandibular jaw

**Figure 1:**
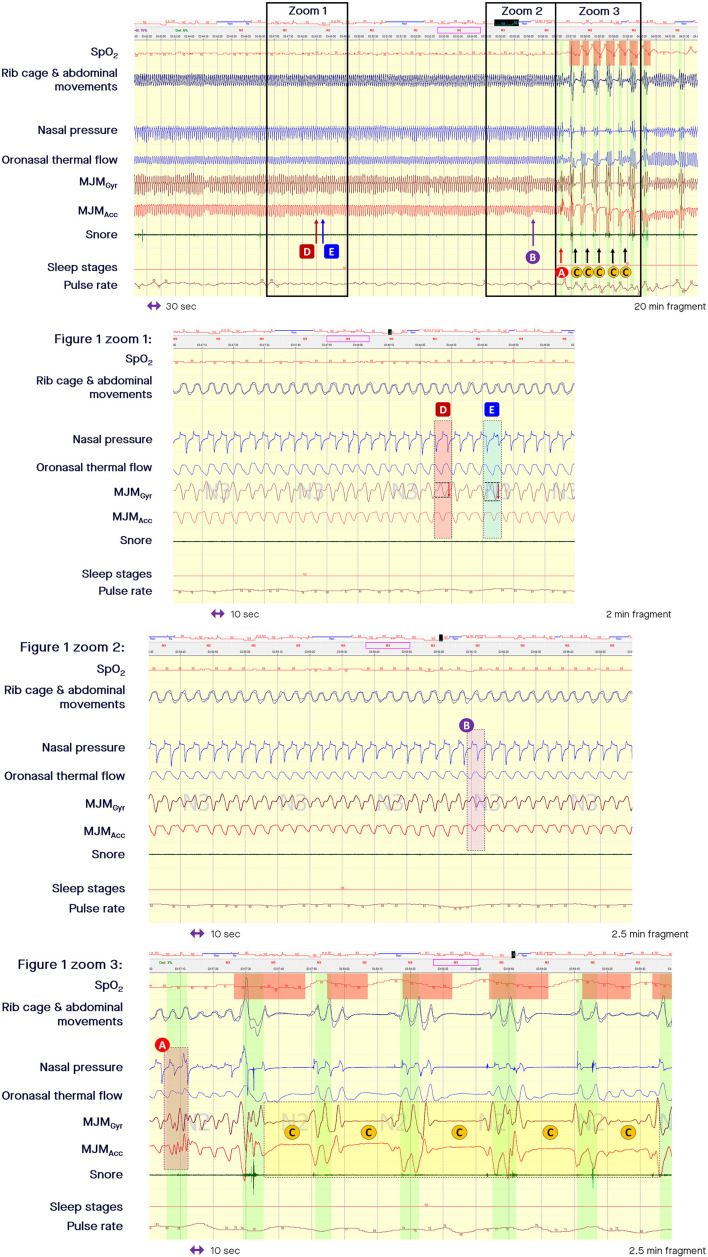
Fragment of polysomnography including the mandibular jaw movements bio-signal showing a long period of respiratory effort-related arousal and then successive episodes of apneas. From the top to the bottom: Oxygen saturation from pulse oximetry; Rib cage and abdominal movements captured by respiratory inductive plethysmography; Nasal pressure canulae; Oronasal thermistor; Mandibular jaw movements (MJM) recorded by the gyroscope (Gyr) and the accelerometer (Acc) of the Sunrise device; Snore monitored by a microphone; Sleep stages; Pulse rate derived from oximetry. The highlighted vertical bars depict the cortical arousals scored with EEG/EMG based rules. **(A)** MJM on arousal, which is significant and of large amplitude due to the related cortical activation intensity. **(B)** Arousals associated with subcortical activation are accompanied by a smaller magnitude of displacement. **(C)** Show the decrease in MJM during central sleep-disordered breathing events. **(D)** Shows that small increases in upper airway inspiratory resistance are accompanied by a small increase in the MJM amplitude. **(E)** Shows how much respiratory effort increases as intraluminal pressure decreases, suctioning the pharyngeal walls during an intra-event negative effort dependence pattern.

During normal sleep the mandibular jaw moves slightly around a fixed position and the mouth is almost closed. However, MJM behind closed lips can be recorded. A physiological displacement of the jaw of only a few 10ths of a millimeter is seen during normal or mildly limited breathing superimposed on the respiratory cycle based on airflow or the respiratory impedance plethysmogaphy (RIP) thoracic and abdominal bands (Figure 1).

## Rationale for evaluating sleep respiratory activity at the mandibular jaw level

Essentially the mandibular jaw plays the role of a lever to stabilize the pharynx. During sleep it is important to ensure upper airway patency in the presence of negative and suctioning pressure inside the upper airway. The latter is countered by the leverage effect of the mandibular jaw that moves a few 10ths of a millisecond before the diaphragm contracts.

By stiffening the pharyngeal walls, the position and movements of the mandible during sleep are important to preserve or restore upper airway patency. These MJM reflect both the central drive and variations in upper airway resistance that typically occur during abnormal respiratory events. As sleep deepens into non-rapid eye movement (NREM) stage 3 (N3) sleep, the upper airway resistance is known to increase, and this is reflected by an increase in the amplitude of MJM (Le-Dong et al., 2021). It can be hypothesized that this increase in MJM amplitude reflects the central drive to the mandibular muscles (depressors and elevators) that act as a lever to stiffen the oral floor (mylohyoid, geniohyoid, and the anterior belly of the digastric muscle) during elevation.

Beyond the longitudinal traction determined by changes in lung volume accompanied by a decrease in the transmural pressure gradient applied to the pharyngeal walls and by an increase in longitudinal airway wall tension, horizontal traction can contribute to upper airway patency via the hyoid bone when this mobile bone is immobilized by tightening of the posterior and inferior muscles (the stylo and mastoido-hyoid and the sterno and crico thyroid-hyoid muscular groups) during a complex coordinated interplay. As suggested by Hollowell et al., the respiratory activity of the mandibular jaw can be described as a co-activation of the depressors/elevators of the mandibular jaw that occurs to finely tune the position of the mandible and ensure upper airway patency during normal sleep (Hollowell and Suratt, 1991). Small increases in upper airway inspiratory resistance are accompanied by small increases in the MJM amplitude, as shown by the arrow D in the zoomed in part of Figure 1.

## Technology for monitoring and recording mandibular jaw movements in the home setting

The first studies validating the clinical use of MJM relied on magnetometry measurements (Nomics, Liège, Belgium; Martinot et al., 2017). More recently, MJM could also be captured using a single point of contact sensor (Sunrise, Namur, Belgium) attached to the patient's chin (in the mentolabial sulcus). This new home sleep apnea test is composed of a coin-sized, tri-axial sensor including a gyroscope and an accelerometer. Jaw displacement is calculated from the rotational speed measured by the gyroscope. This rotational movement is produced by rotation of the mandibular condyle in the temporo-mandibular joint. The position of the mandible resulting from elevation and depression in relation to gravity is provided by the accelerometer.

Recorded MJM data by the sensor are transferred to a smartphone application via Bluetooth for external control. Then, at the end of the night, MJM data are transferred via wireless connection to a cloud-based infrastructure, and data are analyzed with a dedicated machine-learning algorithm that simulates the process of PSG manual scoring. To do so, the algorithm has been trained to recognize stereotypical MJM patterns matching specific PSG signals related to physiological or pathological events (such as sleep stages, arousals, apneas or hypopneas) in order to predict them and compute associated clinical scores delivered in a comprehensive report generated within minutes.

Compared with magnetometry, the tri-axial sensor has some advantages, its ease of use with a single point of contact at the chin level, the higher signal resolution and its ability to record MJM in three dimensions (rather than only vertical movements). Even though there are similarities between the two devices in terms of signal frequency during normal breathing or increased RE, and common specific MJM patterns related to arousals, apnea or hypopnea events, data processing of both devices is different. The machine learning task for the magnetometry bio-signal utilizes only one channel and remains to be further developed and validated, whereas the Sunrise algorithm has been extensively trained and validated over recent years, and utilizes six channels of raw data (x, y, z components of the gyroscope and accelerometer) providing different information at a given time (Pépin et al., 2020; Le-Dong et al., 2021; Martinot et al., 2021, 2022).

Other measurements of related bio-movements also exist, they use surface EMG of the jaw-closing masseter muscles during polysomnography (Kato et al., 2013; Shiraishi et al., 2021). The dissemination of this technique is not possible currently for repeated home sleep apnea testing.

## Clinical applicability of mandibular jaw movement recording to estimate conventional PSG-derived indices

**Figure 2:**
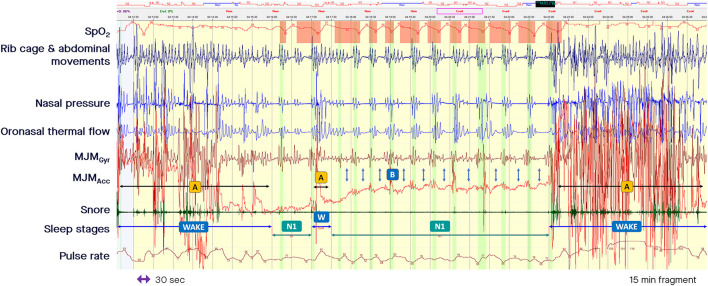
Fragment of polysomnography including the mandibular jaw movements bio-signal showing alternate periods of awakening with non-rapid eye movement light sleep stage. From the top to the bottom: Oxygen saturation from pulse oximetry; Rib cage and abdominal movements captured by respiratory inductive plethysmography; Nasal pressure canulae; Oronasal thermistor; Mandibular jaw movements (MJM) recorded by the gyroscope (Gyr) and the accelerometer (Acc) of the Sunrise device; Snore monitored by a microphone; Sleep stages; Pulse rate derived from oximetry. The highlighted vertical bars depict the cortical arousals scored with EEG/EMG based rules. **(A)** Indicate MJM that are highly variable, often fast, and unpredictable in amplitude while awake. **(B)** Show a decrease of the amplitude in the MJM bio-signal, sometimes reduced to a background noise while asleep. The decrease in central drive can be accompanied by a slight mouth opening.

Measurement of MJM enables accurate sleep time measurement. The shapes, frequencies, and amplitudes of these discrete MJM vary between different sleep stages and breathing disturbances as described by Le-Dong et al. (2021) and Pépin et al. (2020; see the related supplemental documents). When asleep and driven in synchronicity with the respiratory oscillators, mandibular displacements are quite stable compared with awake MJM that are highly variable, often fast, and unpredictable in amplitude and frequency (see arrows A in Figure 2). On arousal, jaw movements are significant and of large amplitude due to the related cortical activation intensity (see arrow A in Figure 1). Arousals associated with subcortical activation are accompanied by a smaller magnitude of displacement (see arrow B in Figure 1). The intensity of these arousals correlates well with the amplitude of the concomitant mandibular jaw displacement, likely due to a related cortico-bulbar reflex. Therefore, the analysis of MJM provides an accurate estimate of sleep/wake states and of the arousal hourly index. Sleep quality metrics showed clinically relevant agreement with manual polysomnographic staging: median [95%CI] differences of −10.25 min [−52.87 to +19.00], 0 min [−23.37 to +6.50], −2.22% [−11.06 to +3.80] for Total Sleep Time, Sleep Onset Latency, and Sleep Efficiency, respectively (Le-Dong et al., 2021).

The use of MJM-derived sleep time instead of recording time as a denominator for calculation of SDB-related indices improves the agreement between the calculated values based on MJM and those visually scored during conventional PSG. The Sunrise derived respiratory disturbances index showed diagnostic capability with ROC AUC of 0.95 (95% CI: 0.92–0.96) and 0.93 (95% CI: 0.90–0.93) for corresponding PSG index of 5 and 15 n/h, respectively (Pépin et al., 2020). Applying the near boundary double labeling method would improve the agreement between Sunrise automated scoring and PSG, by reducing the bias due to inter-human PSG scoring of AHI with an overall agreement (Kappa coefficient) that was 0.80 and 0.86 without and with near boundary double labeling, respectively (Martinot et al., 2022).

**Figure 3:**
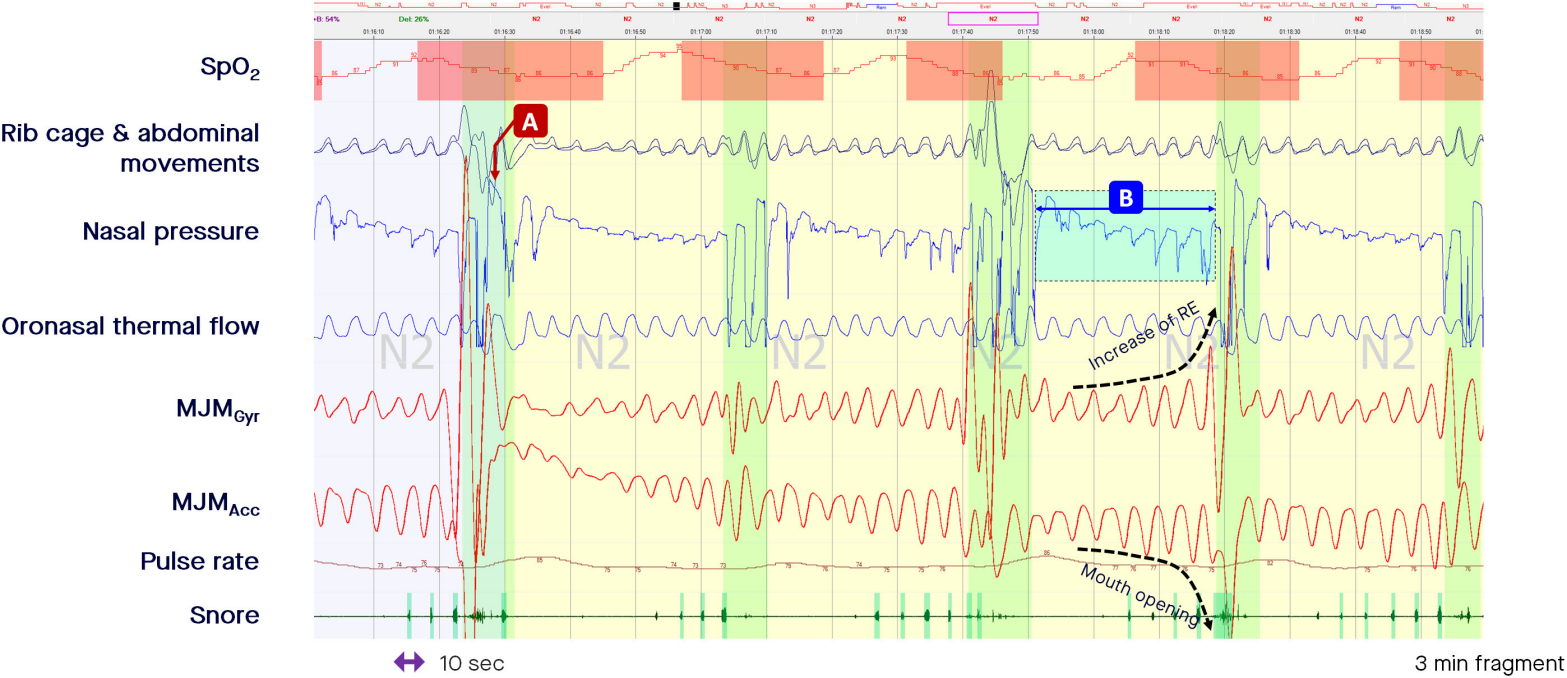
Fragment of polysomnography including the mandibular jaw movements bio-signal to show episodes of “classic” and “drive-dependent” obstructive sleep apnea (OSA). From the top to the bottom: Oxygen saturation from pulse oximetry; Rib cage and abdominal movements captured by respiratory inductive plethysmography; Nasal pressure canulae; Oronasal thermistor; Mandibular jaw movements recorded by the gyroscope (Gyr) and the accelerometer (Acc) of the Sunrise device; Snore monitored by a microphone; Pulse rate derived from oximetry. The highlighted vertical bars depict the cortical arousals scored with EEG/EMG based rules. **(A**, **B)** Represent episodes of “classic” and “drive-dependent” OSA, respectively. The accelerometer channel shows the changes in the mandible position during the period of crescendo pattern: the more the signal is negative, the more the mouth opens until an arousal occurs, characterized by an abrupt and positive displacement of the mandible reflecting the closure of the mouth and peaking the airflow.

During the periods of pharyngeal obstruction that are characteristic of obstructive SDB events, the mouth opens (sometimes with a crescendo pattern, mimicking the typical pattern of crescendo changes in PES during an obstructive apnea or hypopnea) before an arousal occurs, closing the mouth (Figure 3). This is likely due to different carbon dioxide sensitivities between the mandibular jaw depressors and elevators. There is first a more intense phasic recruitment of the depressors leading to mouth opening until an arousal occurs with a peak in airflow coinciding with mouth closure due to a greater recruitment of the elevators. In Figure 3, a decrease in airflow is accompanied by a persistently elevated or increasing rotational speed of the mandible, indicating a persistent high central drive or increasing drive against the upper airway obstruction. During the period of such obstructive episodes different means of MJM amplitudes can be observed possibly as a function of their dominant endotypes (with more or less upper airway muscle gain).

During central SDB events, the amplitude of MJM decreases and the bio-signal can be reduced to a background noise (see arrows C in Figure 1). Due to a decrease in central drive, a slight mouth opening can occur when the elevators cannot sustain the mandible at an upper position. The arrows B in Figure 2 mark a decrease in the MJM bio-signal amplitude, showing a decrease in the trigeminal output. Therefore, changes in MJM amplitude can be used to classify SDB events as central or obstructive based on the ongoing or underlying level of RE. Distribution of MJM amplitude differs significantly between event types: median (95% confidence interval) values of 0.60 (0.16–2.43) for central apnea, 0.83 (0.23–4.71) for central hypopnea, 3.23 (0.72–18.09) for obstructive hypopnea, and 6.42 (0.88–26.81) for obstructive apnea (Martinot et al., 2019; Pépin et al., 2022).

In addition, prolonged periods of sustained inspiratory and/or expiratory effort are well-represented by a progressive increase in MJM amplitude until a relief occurs by way of an arousal (Figure 1). These prolonged periods of RE also called respiratory effort-related arousals (RERAs) can last for more than 1 min, are sometimes accompanied by snoring, and are characterized by a progressive increase in the rotational speed of the mandible. The increased central drive observed through the MJM gyroscopic bio-signal can be stable or unstable, and is marked by several subcortical activations identified by concomitant changes in heart rate, pulse tonometry or pulse transit time when these metrics are evaluated using in-laboratory PSG. Most of the time, these changes are ended by a cortical arousal corresponding to a salient movement of the mandibular jaw to close the mouth.

Hypopnea is the most frequent respiratory event reported during sleep. AASM rules recommend that hypopneas are classified as either obstructive or central depending on the associated RE, reflecting an increase or decrease in the central respiratory command for obstructive and central events, respectively. Correct characterization of the hypopnea sub-type provides information about its origin and contributes to a personalized therapeutic decision-making process (Martinot et al., 2019). As a metric, the overall apnea-hypopnea index (AHI) does not provide accurate clinical risk stratification because it includes both central and obstructive events.

On polygraphy, the presence of elevated RE is assessed by the examination of the dual RIP belt signals (e.g., amplitude and phase shift) and changes in the shape of inspiratory nasal pressure (e.g., flow limitation or plateau aspect) and/or the appearance of a crescendo or stable snoring. Information provided by the RIP belt signals can be misleading in obese patients for instance, since obesity can cause misclassification of obstructive events as central, especially in the unsupervised home setting, and RIP belt signals are prone to fail or disappear (Loube et al., 1999; Masa et al., 2003). A shift phase of the RIP thoracic and abdominal bands belt signals could indicate increased RE and classify the hypopnea as obstructive, although the signal remains difficult to interpret during rapid eye movement (REM) sleep where the tone in the accessory respiratory muscles is lost. In addition, the shape of the nasal pressure signal is altered by mouth breathing and primarily reflects increased upper airway resistance rather than the underlying central drive. A typical flattening waveform strongly suggests an increase in upper airway resistance that could also occur at low levels of RE (Hosselet et al., 1998; Ayappa et al., 2000; Pamidi et al., 2017; Mann et al., 2021). Therefore, a reliable backup signal for RE is required to increase robustness and reliability.

**Figure 4:**
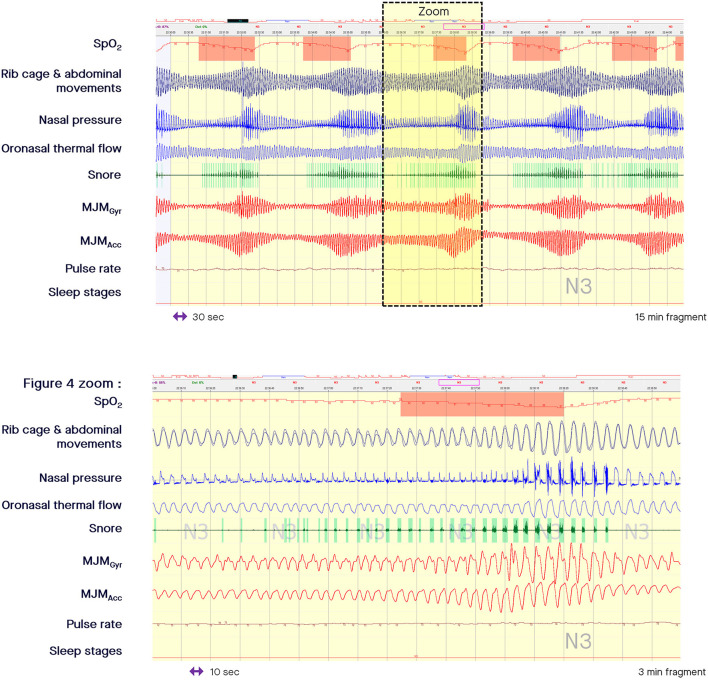
Fragment of polysomnography including the mandibular jaw movements bio-signal showing a period of cyclical breathing. From the top to the bottom: Oxygen saturation from pulse oximetry; Rib cage and abdominal movements captured by respiratory inductive plethysmography; Nasal pressure canulae; Oronasal thermistor; Snore monitored by a microphone; Mandibular jaw movements recorded by the gyroscope (Gyr) and the accelerometer (Acc) of the Sunrise device; Pulse rate derived from oximetry; Sleep stages. In the zoomed in part, the presence of inspiratory flow limitation at the ventilatory peak is clearly evident suggesting that the pharynx is not fully open. Intra-event negative effort dependence patterns are well depicted in the zoomed in part of the figure showing how much respiratory effort increases as intraluminal pressure decreases, suctioning the pharyngeal walls.

When the nasal pressure signal is superposed on the MJM bio-signal at the time of an intra-event negative effort dependence pattern, the increased stimulation of trigeminal motor neurons is clearly associated with a curvilinear decrease in flow signal during the second part of the inspiration with a termination peak. This pattern is well-depicted by an increased peak-to-peak amplitude of the MJM bio-signal (arrow E in the zoomed in part of Figure 1, and zoomed in part of Figure 4). This shows how much RE increases while the intraluminal pressure decreases, suctioning the pharyngeal walls.

The RE measured through MJM monitoring determines the airflow amplitude (central drive) as a function of the residual pharyngeal permeability during the period of obstruction. In the “classic” model of OSA, apneas and hypopneas are characterized by a loss of airway patency precipitating increased central drive, as shown by an increase in the rotational speed of the mandible while the mouth opens more and more until an arousal occurs, closing the mouth and reopening the upper airway, with an associated peak in airflow (arrow A in Figure 3). Until the upper airway reopens, the airflow is abolished or is minimal despite an increase in the central drive. By contrast, the “drive-dependent” model of OSA is characterized by a loss of central drive, promoting dilator muscle hypotonia and, consequently, precipitating respiratory events for many patients. “Drive-dependent” events are depicted by concomitant changes in airflow and MJM amplitude in relation to the degree of pharyngeal obstruction (arrow B in Figure 3; Gell et al., 2022).

During periodic breathing (central SDB), the airflow oscillates between apnea or hypopnea and hyperpnea (where the breathing frequency can accelerate). In Cheyne Stokes respiration (common in patients with heart failure), the airflow follows a characteristic waxing-waning pattern. The Biot's (or ataxic) respiration is characterized by variable changes in flow, random apneas or hypopneas, and no regularity. The specific MJM patterns associated with each change in breathing are detectable based on the level of central drive, as shown in Figure 4. In the zoomed in part of Figure 4, the presence of inspiratory flow limitation at the ventilatory peak, and the presence of snoring strongly suggest that the pharynx is not fully open.

## Conclusion

MJM provide a robust and comprehensive bio-signal to detect SDB. During normal periods of breathing during sleep, the mandible (or lower jaw) moves slightly around a fixed position. The shapes, frequencies, and amplitudes of these discrete MJM vary between different sleep stages and breathing disturbances. Chemoreceptors and upper airway pressure sensors inform the brain about breathing disturbances. In response, an increase in the central motor drive will recruit and stiffen the pharyngeal muscles so that normal breathing resumes. Many of these muscles are attached to the mandible. Therefore, contracting or relaxing these muscles generates discrete and informative MJM. The ability of MJM to accurately measure RE during sleep has been confirmed during simultaneous, synchronized in-laboratory PSG (Pépin et al., 2022).

Overall, the measurement of MJM provides the clinician with deep insights into the work of breathing, including intra-event negative effort dependence patterns and differentiation between central and obstructive SDB events, including central hypopneas. Essentially, monitoring MJM is like having a probe in the brainstem to observe the regulating activity of the brain during sleep. This provides a new, comprehensive, reliable, validated and objective way of detecting and characterizing SDB.

## Author contributions

Both authors contributed to manuscript preparation, revision, read, and approved the submitted version.
